# Acute Neurological Involvement after Donor Lymphocyte Infusion for Post-Transplant Viral Infection: The Same Pattern of Novel Cancer Immunotherapy-Related CNS Toxicity?

**DOI:** 10.3390/ijms23073553

**Published:** 2022-03-24

**Authors:** Annalisa Marcuzzi, Erika Rimondi, Elisabetta Melloni, Floriana Zennaro, Aurelio Sonzogni, Sara Leo, Natalia Maximova

**Affiliations:** 1 Department of Translational Medicine, University of Ferrara, 44121 Ferrara, Italy; annalisa.marcuzzi@unife.it; 2 Department of Translational Medicine and LTTA Centre, University of Ferrara, 44121 Ferrara, Italy; erika.rimondi@unife.it; 3 Studio Biomedico Trieste Centro, Policlinico Triestino, 34137 Trieste, Italy; fzennaro@mac.com; 4 Ospedale Papa Giovanni XXIII, Department of Pathology, 24127 Bergamo, Italy; asonzogni@asst-pg23.it; 5 Department of Environmental Sciences and Prevention, University of Ferrara, 44121 Ferrara, Italy; sara.leo@unife.it; 6 Bone Marrow Transplant Unit, Institute for Maternal and Child Health-IRCCS Burlo Garofolo, 34137 Trieste, Italy; natalia.maximova@burlo.trieste.it

**Keywords:** stem cell, transplantation, immunotherapy, viral infection, cytokines

## Abstract

Early post-transplant is the critical phase for the success of hematopoietic stem cell transplantation (HSCT). New viral infections and the reactivations associated with complete ablation of the recipient’s T-cell immunity and inefficient reconstitution of the donor-derived system represent the main risks of HSCT. To date, the pharmacological treatments for post-HSCT viral infection-related complications have many limitations. Adoptive cell therapy (ACT) represents a new pharmacological strategy, allowing us to reconstitute the immune response to infectious agents in the post-HSC period. To demonstrate the potential advantage of this novel immunotherapy strategy, we report three cases of pediatric patients and the respective central nervous system complications after donor lymphocyte infusion.

## 1. Introduction

Hematopoietic stem cell transplantation (HSCT) has become a well-established treatment for various malignant and non-malignant disorders originating from the hematopoietic system [1].

However, the early post-transplant period is a critical and crucial phase for the success of HSCT.

The first months after HSCT are characterized by a high incidence of viral infections and reactivations due to the complete ablation of the recipient’s T-cell immunity and slow reconstitution of the donor-derived immune system [2]. Viral infections are mainly caused by the reactivation of latent viruses, such as cytomegalovirus (CMV), Epstein–Barr virus (EBV), adenovirus (ADV), herpes simplex virus (HSV) and varicella-zoster virus (VZV) [3,4]. Due to recent advances in CMV detection and treatment options, the incidence of CMV disease has decreased. Despite better pre-emptive management of CMV reactivation/infection, CMV-seropositive patients continue to experience poorer outcomes than CMV-seronegative patients through increased non-relapse mortality and decreased overall survival [5]. EBV-related post-transplant lymphoproliferative disorders (PTLDs) are a group of severe complications of HSCT and solid organ transplantation. The mortality rate, approximately 33%, remains high despite the therapeutic approaches having been significantly improved with the use of rituximab and adoptive cell therapy [6]. Adenoviruses are responsible for a significant percentage of severe complications in allogeneic HSCT recipients, in particular disseminated disease. In the pediatric population, the mortality associated with adenovirus is more severe compared to adults, with reported rates as high as 82% [7]. Current pharmacological options for the treatment of post-HSCT virus infection-related complications have several limitations, including dose-limiting end-organ toxicity, particularly myelotoxicity and lack of efficacy due to primary or secondary resistance [8].

Adoptive cell therapy (ACT) involves the infusion of virus-specific T cells from a donor to recipient and is a new possibility to rapidly reconstitute immune responses to infectious agents in the post-HSCT period. Virus-specific T cells, after in vivo expansion, can mediate long-term infection control. ACT offers several potential advantages over pharmacotherapy, including the specificity of cytotoxic responses to pathogens and establishing long-term T-cell memory while avoiding some of the organ toxicities associated with antiviral drugs [9].

We report three cases of unmanipulated lymphocyte infusion for a post-transplant life-threatening viral infection, using related donors who developed antibodies against the same viral strain. In our study, the patients affected by non-malignant diseases were subjected to myeloablative conditioning. To avoid rejection, these patients needed to undergo more heavy pre-transplant immunosuppression than oncological recipients. Moreover, all three patients described in the case series were fully lymphodepleted, agreeing with the transplantation timing. A specific focus of our manuscript is on describing the central nervous system complications after donor lymphocyte infusion in these three pediatric patients, not yet described before.

## 2. Case Reports

### 2.1. Case 1

A four-year-old Caucasian boy, affected by homozygous sickle cell anemia (78.2% of HbS), underwent an allogeneic HSCT from a sibling donor, preceded by myeloablative conditioning. The stem cell source was bone marrow, and 7.5 × 10^8^ total nuclear cells/kilogram of body weight were infused at day 0. The anti-rejection prophylaxis was performed with tacrolimus.

Three days before the bone marrow harvest, the donor repeated virological tests required by the Italian Bone Marrow Donor Registry, including the serological test for EBV, negative at the initial screening. Unfortunately, due to technical problems, the results of the antibody panel were released with a delay and we overlooked them. At day +30, we detected elevated liver enzymes and the presence of a high EBV-DNA plasma load (5.9 × 10^6^ copies/mL) by real-time fluorescence quantitative PCR. No signs of skin or intestinal graft-versus-host disease (GVHD) were present. Fever appeared a few days later, with no increase of inflammation markers. Flow cytofluorimetric analysis demonstrated the presence of monoclonal B-cell expansion (30% CD19, 195 cells/mm^3^) with a normal kappa:lambda ratio. The patient was completely T-cell-depleted, with the CD3 count at only 1% (7 cells/mm^3^) and a high CD16/56 (NK) level (68%, equal to 448 cells/mm^3^). Thoracic MRI confirmed a post-transplant lymphoproliferative disorder (PTLD), showing the presence of diffusion restriction areas corresponding to the right paratracheal lymph node chains, associated with a left peribronchial area presenting a similar scenario. The first rituximab administration led to a null B lymphocyte count, with no effect on the viral load and fever. Lymphocytes’ analysis revealed a significant colonization of NK lymphocytes by EBV. 

**Figure 1:**
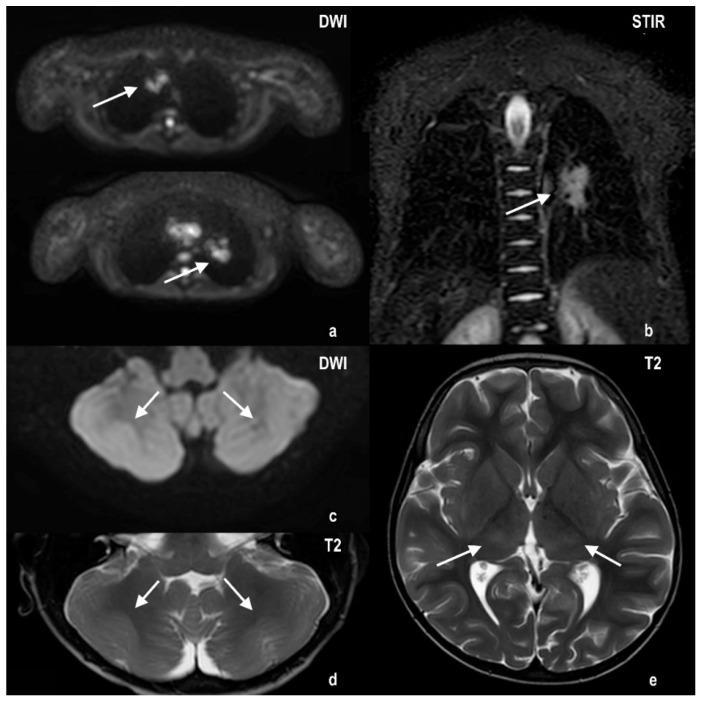
Thoracic MRI shows DWI restriction areas corresponding to the right paratracheal lymph node chains, associated with a left peribronchial area presenting a similar aspect (panel (**a**): axial DWI; panel (**b**): coronal STIR). Cerebral MRI shows bilateral symmetric cerebellar cortex swelling and T2 hyperintensity, with DWI (b1000). Restricted diffusion from cytotoxic vasogenic edema can be seen (panels (**c**,**d**): cerebellar DWI and T2, respectively), along with T2 hyperintensity of posterior thalami (panel (**e**): axial T2).

The source of the EBV infection was certainly his donor. When the bone marrow was harvested, his sister presented serum markers of a recent EBV infection, and the patient, meanwhile, developed EBV-specific immunity. At this point, the only suitable therapeutic strategy was a donor lymphocyte infusion (DLI). Thus, on day +43, we infused 5.9 × 10^7^/kg of the patient’s weight of the donor’s CD3 lymphocytes, leading to two log reductions of the EBV load in 48 h. Unfortunately, the EBV load reduction was accompanied by a progressive worsening of the patient’s general condition, with asthenia leading to lethargy, and later, to partial and generalized seizures four days after the DLI. The brain MRI showed bilateral symmetric cerebellar cortex swelling and T2 hyperintensity, with diffusion-weighted imaging (DWI) (b1000) revealing restricted diffusion from a cytotoxic vasogenic edema, and T2 and fluid-attenuated inversion recovery (FLAIR) of the hyperintensity of the posterior thalami, with restricted DWI diffusion (Figure 1).

**Figure 2:**
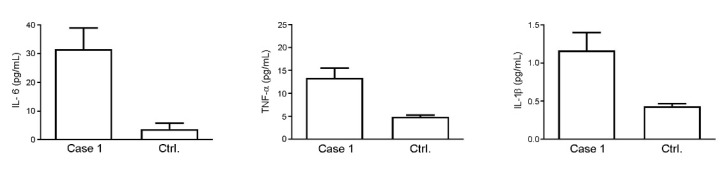
Analysis of levels of IL-6, TNF-α and IL-1β in the serum of Case 1 compared to control donors. Values are expressed as pg/mL.

The thoracic MRI showed a decreased diffusion restriction of right paratracheal lesions and reduced volumes of the left peribronchial lesions. The electroencephalography (EEG) demonstrated the presence of diffuse slow (sub delta) and mean width activity and the absence of any stable asymmetry or epileptic focus. To rule out central nervous system (CNS) EBV-related involvement, a lumbar puncture was performed. The cerebrospinal fluid (CSF) was acellular with normal protein and glucose levels. All viral CSF determinations were negative. Therefore, we also carried out a CSF cytokine assessment. The data showed a high level of IL-6 (75.19 ± 15.89 pg/mL) compared to control values reported in the literature data (Table 1), while the TNF-α (5.21 ± 0.50 pg/mL) and IL-1β (0.32 ± 0.02 pg/mL) did not seem to differ from the reference values reported in Table 1. The cytokines were also evaluated in the patient’s serum collected at the same time as CSF (Figure 2), showing higher levels of TNF-α and IL-6 when compared to control donors (13.37 ± 2.13 vs. 4.87 ± 0.38 and 31.61 ± 7.41 vs. 3.73 ± 2.05 pg/mL, respectively), and an IL-1β value at the lower limit of detection (1.17 ± 0.24 pg/mL).

Immune effector cell-associated neurotoxicity syndrome (ICANS) was suspected. We treated the patient with a high dose of infliximab (10 mg/kg), achieving complete resolution of neurological symptoms within a few hours.

Two weeks after the DLI, at day +57, cytofluorimetric evaluation showed rapid immune reconstitution, with 2020 total lymphocyte/mm^3^, 1495 CD3/mm^3^, 323 CD4/mm^3^ and EBV clearance also achieved.

### 2.2. Case 2

A seven-year-old girl with acute lymphoblastic leukemia, diagnosed at four years old, with several SNC relapses, underwent an allogeneic HSCT from a sibling donor, then relapsed two months later. After a short course of rescue chemotherapy, the girl was undergoing haploidentical HSCT from her father.

The early post-transplant period was uneventful, and she was discharged at day +39 after rapid and complete engraftment and leukemic remission. The patient’s immunological status was still profoundly depressed, with only 120 total lymphocytes/mm^3^ and the absence of T lymphocytes in her peripheral blood. For this reason, home isolation was strongly recommended. 

One week later, the girl was readmitted to our center with a high fever, diffuse skin rash and profuse watery diarrhea. All blood and stool microbiological and viral investigations were negative except for detecting human adenovirus (HAdV) DNA both in the blood (4.1 × 10^5^ copies/mL) and in the stool. Antiviral treatment with cidofovir was immediately initiated. 

A few days before her infectious event, some relatives had a self-resolved pharyngoconjunctival fever. Suspecting a domestic origin of the infection, we performed HAdV research on the relatives who had a fever a few days earlier. The father’s throat swab was positive for HAdV. Genotype analysis typed the HAdV as subgroup C, type 1, which was the same as the patient.

**Figure 3:**
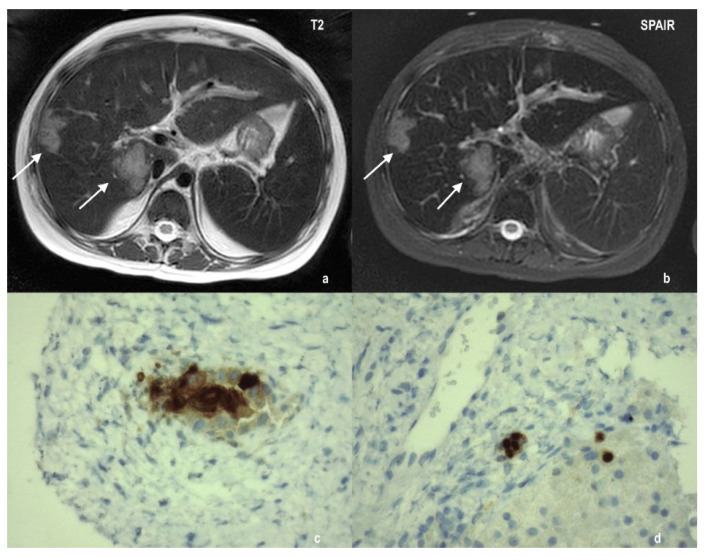
Radiological and histological presentation of adenoviral hepatitis. Liver MRI shows multiple parenchymal lesions (indicated by the white arrows), hyperintense in T2 weighted sequences (panel (**a**): axial T2; panel (**b**): axial SPAIR). Immunohistochemical staining for adenovirus makes evident the presence of HAdV in hepatocytes (panels (**c**,**d**)).

Despite treatment with cidofovir, the girl’s general condition deteriorated with the persistence of high fever and conversion of watery diarrhea to frankly hemorrhagic. Subsequently, the patient developed severe liver dysfunction, and the presence of multiple parenchymal lesions was documented by hepatic computed tomography and MRI examination. A liver biopsy demonstrated the presence of HAdV in hepatocytes’ cytoplasm (Figure 3).

In the meantime, the father completely recovered from the infectious event and the second throat swab was negative. Considering that disseminated HAdV disease in severely immunocompromised patients is often fatal [23], we decided to take advantage of the virus-specific immunity obtained by the father following the recent infection. Four days later, the father’s lymphocytes were collected, and the girl received 5.1 × 10^6^ CD3/kg of her body weight.

**Figure 4:**
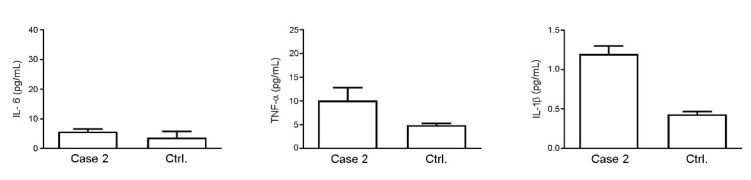
Analysis of levels of IL-6, TNF-α and IL-1β in the serum of Case 2 compared to control donors. Values are expressed as pg/mL.

The fever completely resolved in a few days, while the hemorrhagic enterocolitis and viral hepatitis promptly improved during the first week post-DLI. In addition, blood and stool HAdV clearance was achieved eight days after DLI. However, signs of acute neurological injury and fever appeared the day after the virus clearance was documented. She appeared initially somnolent and confused, with a fluctuating level of consciousness. A few hours later psychomotor agitation, hallucinations and prominent tremors arose. To rule out CNS disease relapse or infection, a lumbar puncture was performed. The CSF analysis revealed acellular fluid with a slightly elevated protein level (50 mg/dL), without evidence of blast cells in flow cytometry. HAdV DNA tests and those for other pathogens were all negative. Cytokine CSF evaluation showed that for this patient, the IL-6 level was a little higher (38.41 ± 9.71 pg/mL) than the literature data (Table 1), while the TNF-α (4.43 ± 1.13 pg/mL) and IL-1β (0.39 ± 0.10 pg/mL) levels were in the range of the control values. Serum analysis of the same cytokines showed higher levels of TNF-α when compared to control donors (10.01 ± 2.70 vs. 4.87 ± 0.38), while the IL-6 did not depart too much from control values (5.75 ± 0.85 pg/mL vs. 3.73 ± 2.05). The IL-1β level was, even in this case, at the lower limit of detection (1.20 ± 0.1 pg/mL) (Figure 4).

The EEG findings were diffuse, slowing with triphasic waves of generally 2–3 Hz. Brain MRI FLAIR hyperintensities were seen in the bilateral thalami, consistent with vasogenic edema. At the same time, flow cytometry analysis of the peripheral blood sample documented an increase of the total lymphocyte counts (1100 cell/mm^3^) by >600 CD3/mm^3^ and >200 CD4/mm^3^.

Immediately after ruling out CNS infection or leukemia relapse, high-dose infliximab (10 mg/kg) was administered, leading all neurological symptoms to subside within hours.

### 2.3. Case 3

A 12-month-old baby, affected by autosomal recessive osteopetrosis (ARO), in the advanced stage of disease, was admitted to our Bone Marrow Transplant Unit. The detection of a homozygous g.9909 G>A mutation in exon 14 of the *TCIRG1* gene confirmed the ARO form of the disease. An unfavorable prognosis, with limited probability of success of HSCT and high risks of transplant-related mortality associated with the advanced stage of disease, was communicated to the parents. Moreover, a consent agreement form was signed by the parents, and a haploidentical transplant was immediately scheduled. At the time of the pre-transplant evaluation, the only immediately available donor was his mother. Serological tests, performed to determine donor eligibility, confirmed on three occasions the presence of the IgG antibodies anti-viral capsid antigen (anti-VCA IgG), anti-Epstein-Barr virus nuclear antigen (anti-EBNA IgG) and anti-early antigen (anti-EA IgG). However, our anti-VCA IgM and PCR for plasma EBV-DNA were repeatedly negative. We evaluated the frequency of EBV-specific cytotoxic T lymphocytes (CTLs) in the peripheral donor blood with an IFN-γ ELISPOT assay [11], showing a high level of EBV-specific CTL, equal to 126 spot-forming units (SFUs)/10^5^ cells.

The patient underwent neurosurgery for suboccipital decompression and cervical laminectomy, followed by the first haploidentical HSCT. Initial engraftment was achieved, but rejection occurred two months later. A second haploidentical transplant from the same donor was performed ten weeks after the onset of rejection, which led to permanent engraftment of the donor’s hematopoietic stem cells. 

On day +18, after a few days of high fever, we documented, for the first time, the presence of an elevated EBV-DNA plasma load (4.6 × 10^4^ copies/mL). In addition, an abdominal ultrasound evaluation evidenced multiple hypointense areas in the hepatic parenchyma. Flow cytofluorimetric analysis demonstrated complete T- and B-lymphocyte depletion: CD3 0.7% (3 cells/μL) and CD19 0.5% (2 cells/μL). The peripheral blood lymphocytes were mainly CD16+/56+ (98%, equal to 437 cells/μL), with a high rate of EBV colonization. The hepatic parenchyma biopsy specimen was not diagnostic of a PTLD, while Epstein-Barr virus-encoded small RNA (EBER) assessment of the biopsy sample was positive. Meanwhile, the EBV viral load increased to 9.2 × 10^5^ copies/mL. While waiting for the collection of maternal lymphocytes, the child received one dose of rituximab with a limited viral load response. Four days later, we infused the first DLI at a 0.7 × 10^6^ CD3/kg dose, followed by a second DLI two weeks later. After the first DLI, the fever subsided rapidly, and the viral load was halved. Thus, viral clearance was achieved along with the disappearance of liver lesions after the second DLI.

Unfortunately, two days after the second DLI, we noted progressive cognitive impairment of the child, tremors and somnolence, followed by a generalized seizure. Electroencephalography evidenced diffuse generalized slowing without any stable asymmetry or epileptic focus. The brain MRI showed T2 hyperintensity and swelling, associated with diffusion restriction, of the bilateral thalami. 

Unfortunately, it was not possible to obtain an MRI image because the patient, suffering from ARO, presented severe skeletal alterations, and for this reason, the radiological picture would have been superimposed on the neuronal damage caused by the viral infection, preventing a correct visualization with imaging methods.

**Figure 5:**
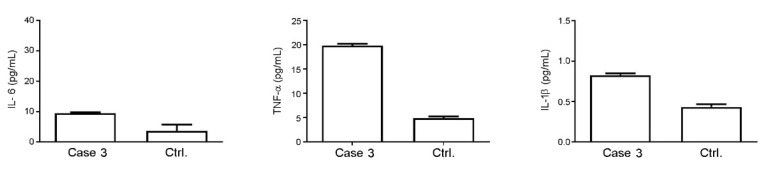
Analysis of levels of IL-6, TNF-α and IL-1β in the serum of Case 3 compared to control donors. Values are expressed as pg/mL.

CSF examination revealed acellular fluid, with normal protein and glucose concentrations, absence of any infectious agents and unusually high levels of IL-6 (99.87 ± 6.77 pg/mL) compared to the literature data (Table 1), while the TNF-α (4.76 ± 1.96 pg/mL) and IL-1β (0.19 ± 0.03 pg/mL) levels were comparable to those of control cases reported in the references of Table 1. As shown in Figure 5, serum analysis revealed that the TNF-α and IL-6 levels were higher than those of control patients (19.83 ± 0.37 vs. 4.87 ± 0.38 and 9.57 ± 0.25 vs. 3.73 ± 2.05 pg/mL, respectively), and that the IL-1β was near to the lower limit of detection (0.82 ± 0.03 pg/mL).

DLI-related neurotoxicity was suspected, and the child was treated with a high dose of infliximab (10 mg/kg), leading to the rapid resolution of neurological symptoms.

One month after the second DLI, the baby’s T-lymphocyte reconstruction was almost complete. Furthermore, we documented a high frequency of EBV-specific T lymphocytes in the peripheral blood (205 SFU/10^5^ cells).

## 3. Discussion

Cytokine release syndrome (CRS) and immune effector cell-associated neurotoxicity syndrome (ICANS) are immune-mediated toxicities characterized by overexpression and hyper-activation of pro-inflammatory cytokines, within which TNF-α plays a key pathogenetic role [24,25,26]. 

TNF-α is a cytokine equipped with pleiotropism that produces several immunological and inflammatory responses in different local areas of the body (such as the endothelial system) and at the systemic level (liver, bone marrow and nervous system) [27]. As early as 1997, Lucas et al. showed that the overexpression of TNF-α by astrocytes or neurons is sufficient to trigger a neurological disease characterized by ataxia, convulsions and paresis with histopathological characteristics of chronic inflammation and degeneration of the white substance in the CNS [28].

Therefore, both soluble and transmembrane forms of TNF-α can play important roles in vivo in the pathogenesis of inflammation and demyelination of CNS.

Given its crucial role in the inflammatory process, this molecule has been the therapeutic target of specific pharmacological treatments, the most significant example of which is infliximab, the use of which is approved for pathologies such as rheumatoid arthritis and chronic inflammatory bowel diseases (Crohn’s disease and ulcerative colitis) [29,30,31].

Recent literature data have also shown that TNF-α inhibition is an effective neuroprotective strategy against neurodegeneration in preclinical studies on in vivo models. In particular, this result has been confirmed in neurodegenerative diseases such as Alzheimer’s and multiple sclerosis, characterized by serious cognitive disabilities. Under these conditions, the new neuroprotective pharmacological strategy is based on the inhibition of TNF-α, which, according to the results collected, seems to have a protective effect on patients with mild cognitive deterioration by counteracting inflammation [32,33,34].

Research into the TNF-α inhibition mechanism within the inflammatory process has allowed us to learn in greater depth the relationship that exists between this cytokine and other molecules involved in inflammation such as IL-1β and IL-6 [35,36,37].

This interconnection was confirmed in studies that demonstrated the ability of TNF-α and IL1β to modulate neuronal plasticity in neuroinflammation [38], as well as in studies in which TNF-α, IL 6 and IL1β overexpression was identified in Alzheimer’s as a factor promoting neurodegeneration [39,40].

The effectiveness of the inhibitory effect of TNF-α in limiting neuroinflammation was also evident in the three cases presented in this study. All patients had central nervous system complications associated with viral infections in the first post-hematopoietic stem cell transplantation (HSCT) period. 

According to these observations, the main focus of this study was describing a new pharmacological strategy for adoptive cell therapy to counteract the overexpression of cytokines, caused by the hyperimmune response, thereby reducing CNS dysfunction.

For these patients, adoptive cell therapy was applied to treat post-transplant life-threatening viral infections, using related donors who developed antibodies against the same strain of the virus.

Complications of the central nervous system became evident, with MRI of the brain showing hyperintensity of T2 and swelling, associated with diffusion restriction, of the bilateral thalami. This inflammatory condition was also confirmed by the cytokine profile traced in the CFS. We should further note that this inflammatory trend was found at the systemic level in the sera of the three patients, where the levels of the cytokines evaluated (IL-1, IL6, TNF-α) were higher compared to control donors, although no statistical analysis was possible for the limited number of samples.

According to this common condition among patients, the anti-inflammatory effect of inhibition of TNF-α by infliximab was relevant since, in all three cases, it was able to restore general homeostasis.

This effect can probably be explained by the mechanism of crosstalk between these three molecules, thanks to which we could hypothesize that the inhibitory effect exerted on TNF-α can induce a progressive intracellular signal cascade that also involves the other two cytokines. Of course, we are aware that this hypothesis still needs further study and an evidence base, but the clinical records of these three patients lend support to the notion of a close relationship between the inflammatory molecules, even when resulting from the activation of different pathways, suggesting that this mechanism could be a useful tool to identify new therapeutic targets.

## 4. Methods

### 4.1. Patients

Three pediatric patients (aged 1–7 years) treated in the Bone Marrow Transplant Unit of IRCCS Burlo Garofolo, Trieste, Italy were selected to participate in the present retrospective study. All study patients had undergone allogeneic HSCT preceded by a myeloablative conditioning regimen [41]. All the real-time quantitative PCR analyses to detect viral infection and for lymphocyte cytofluorimetric phenotyping were performed following routine diagnostic protocols.

After the parents signed their informed consent, data of interest (i.e., age, weight, clinical chemistry values, therapies, therapeutic drug monitoring values, etc.) were collected in a completely anonymized fashion to protect the patients’ privacy.

The planning and the execution of this study were approved by the Ethics Committee of the Institute for Maternal and Child Health, IRCCS Burlo Garofolo [42].

The selection of control donors, for ethical reasons, was limited to infants and young children needing to undergo medically indicated peripheral venous blood sampling before elective surgical interventions or within the scope of diagnostic procedures. Moreover, we excluded subjects affected by an acute or chronic infectious disease.

### 4.2. Cytokines’ Evaluation

Patients’ sera were tested to evaluate the following cytokines: IL-1β, IL-6 and TNF-α, using the human cytokine BioPlex assay (BioRad Laboratories, Milan, Italy), a magnetic bead-based multiplex kit. Serum samples used for the immunoassay test were frozen and thawed only once. Cytokines’ evaluation was performed according to the manufacturer’s instructions using a Bio-Plex 200 instrument equipped with the Bio-Plex Manager software, with a five-parameter non-linear regression formula used to compute the sample concentrations from the standard curves. Unfortunately, with us having only two serum samples from each patient, no statistical analysis could be carried out.

### 4.3. MRI and Histological Analysis

Thoracic, abdomen and brain magnetic resonance imaging (MRI) were performed with a Philips Ingenia scanner at 1.5T (Eindhoven, Netherland). Axial diffusion-weighted imaging (DWI) and coronal short tau inversion recovery (STIR) free-breathing sequences were used for lung evaluation, axial T2 and DWI sequences for brain imaging and axial T2 and spectral adiabatic inversion recovery (SPAIR) for liver parenchyma assessment. 

Liver needle sampling was formalin-fixed and paraffin-embedded. Serial histological sections were taken and stained routinely by hematoxylin and eosin, trichrome stain and PAS stain. Adenovirus was sought using an immunohistochemical procedure with Adenovirus (20/11 & 2/6) Mouse Monoclonal Antibody (Cell Marque, Merck KGaA, Darmstadt, Germany).

## 5. Conclusions

In this paper, we have presented three cases of patients with clinical manifestations that we can identify as belonging to CRS, and due to the neurological symptoms, to ICANS. Several pieces of evidence support the involvement of pro-inflammatory cytokines in the pathogenesis of CRS/ICANS, characterized by high levels of circulating pro-inflammatory cytokines, acute systemic inflammatory manifestations and secondary organ involvement [43]. In this context, our results have demonstrated that a cytokine blockade was able to relieve neurological manifestations in three patients who exhibited neuroinflammation associated with viral infections after HSCT. The use of infliximab, a known TNF-α inhibitor, led to a complete remission of neurological symptoms. This observation could be attributed to the crosstalk between the cytokines involved in neuroinflammation. We are aware that other studies are necessary to confirm our hypothesis based on clinical data, but our results could be useful to hypothesize new therapeutic strategies to counteract the CRS/ICANS effects.

## Figures and Tables

**Table 1:** Reference values for IL-1β, IL-6 and TNF-α levels in CSF of control subjects of different studies indicated.

Subjects	Cytokine	CSF Control Values (pg/mL)
Koeken, V.A.C.M. et al. [10]	IL-1β	0.19 (mean)
Šumanović-Glamuzina, D. et al. [11]	IL-1β	0.05 (median)
	IL-6	7.00 (median)
	TNF-α	16.20 (median)
Krebs, V.L. et al. [12]	IL-1β	0.0 (median)
	IL-6	5.1 (median)
	TNF-α	0.0 (median)
Lindqvist, D. et al. [13]	IL-1β	0.07 (mean)
	IL-6	0.64 (mean)
	TNF-α	0.13 (mean)
Liu, Q. et al. [14]	IL-1β	0.21 (median)
	IL-6	5.71 (median)
	TNF-α	3.32 (median)
Is, M. et al. [15]	IL-6	28.6 (mean)
	TNF-α	14.4 (mean)
Pinto Junior, V.L. et al. [16]	IL-6	0.06 (median)
Lee, K.Y. et al. [17]	IL-6	8.73 (median)
Nagashima, H. et al. [18]	IL-1β	0.35 (mean)
	IL-6	4.58; 5.14 (mean)
	TNF-α	undetectable
Schwieler, L. et al. [19]	IL-6	1.50 (median)
Stelmasiak, Z. et al. [20]	IL-6	0.87 (mean)
Alexander, G.M. et al. [21]	IL-1β	0.02 (mean)
	IL-6	1.32 (mean)
	TNF-α	0.11 (mean)
Pilotto, A. et al. [22]	IL-1β	0.27 (median)
	IL-6	1.05 (median)
	TNF-α	0.17 (median)

## Data Availability

The datasets used and/or analyzed during the current study are available from the corresponding author on reasonable request. The data are not publicly available due to the impracticality of public access.
